# Acute kidney injury associated with minimal change disease in systemic lupus erythematosus: a case report

**DOI:** 10.1186/1752-1947-8-422

**Published:** 2014-12-13

**Authors:** Xin Wei, Ying Wang, Luxia Tu, Hongping Wan, Qinkai Chen

**Affiliations:** 57 Department of Nephrology, The First Affiliated Hospital, Nan Chang University, YongWai street 17#, Nanchang city, Jiangxi province 330006 China; 58 Department of Pathology, The First Affiliated Hospital, Nan Chang University, YongWai street 17#, Nanchang city, 330006 China

**Keywords:** Acute kidney injury, Minimal change disease, Systemic lupus erythematosus

## Abstract

**Introduction:**

In systemic lupus erythematosus, acute kidney injury is usually associated with severe lupus nephritis and rarely associated with other glomerular diseases.

**Case presentation:**

We recently encountered a patient with acute kidney injury that was associated with minimal change disease in systemic lupus erythematosus. A 26-year-old Chinese woman who had a history of systemic lupus erythematosus presented with nephrotic syndrome and acute kidney injury. She fulfilled four of the American College of Rheumatology criteria for the classification of systemic lupus erythematosus. However, a renal biopsy revealed that there were no glomerular abnormalities or deposition of immune complex. Her generalized edema disappeared, and her high serum creatinine level decreased to normal after prednisolone therapy.

**Conclusion:**

Though the relationship between lupus and minimal change disease is still not defined, the possibility of systemic lupus erythematosus combined with minimal change disease must be differentiated in patients with lupus and severe proteinuria.

## Introduction

Systemic lupus erythematosus (SLE) is an autoimmune disease characterized by pathogenic autoantibodies and deposition of immune complexes in various organs. Lupus nephritis (LN) is caused by immune complex deposition in the glomerulus [1].

Diffuse proliferative LN and membranous LN are two predominate pathologic findings in LN. SLE combined with minimal change disease (MCD) has rarely been reported. In this report, we describe the case of a patient with SLE who had acute kidney injury (AKI) caused by MCD. She was treated successfully with steroids with a follow-up duration of 2 years.

## Case presentation

A 26-year-old Chinese woman with a history of SLE of 10 years presented to our hospital. Her initial symptoms 10 years before her visit had been arthritis, malar erythema and prolonged fever. The following were her laboratory investigation results at that time: antinuclear antibody (ANA) titer, 1:160 (+); anti-Smith (anti-Sm) antibody, +; urinary protein level, 3+; hematuria, −; complement 3 value, 0.36g/L. Her renal function test and hematologic evaluation results were within normal ranges. A renal biopsy was not conducted at that time. Prednisolone 1mg/kg/day (40mg/day) was prescribed first and then gradually tapered to 5mg/day over the course of 12 months. The patient had complete remission of signs and symptoms of SLE after the corticosteroid treatment. She took prednisolone 5mg/day as maintenance therapy during the next 10 years.

When she was admitted to our hospital, she had a rapidly progressive edema and oliguria (urine output was about 250ml/day). Her physical examination revealed that she had mild edema of the face and legs. She had not taken any drugs other than prednisolone within the preceding 6 months. Her blood pressure level was 108/71mmHg. Her heart and lungs were normal. No liver, spleen or lymph node enlargement was found. The following were the results of her laboratory investigations: urinary protein, 3+; 24-hour urinary protein, 4.16g/day; hematuria, −; blood urea nitrogen, 21.0mmol/L; creatinine, 455μmol/L; albumin, 22.8g/L; total cholesterol, 5.26mmol/L; triglycerides, 1.45mmol/L; hemoglobin, 10.3g/dl; white blood cell count, 3.08×10^9^/L; red blood cell count, 3.53×10^12^/L; platelets, 81×10^9^/L; ANA, 1:320 (+); complement 3, 0.60g/L; anti-DNA antibody–negative; anti-Sm antibody–negative; anti-ribonucleoprotein-negative; hepatitis B surface antigen–negative; anti-hepatitis C virus–negative; and anti-HIV-negative. Her chest digital radiography and abdominal ultrasonography results were normal. She did not have any other comorbid conditions or recent infections. Within just 1 week, the patient’s symptoms got worse, her edema became aggravated, her urine output decreased to 50ml/day and her serum creatinine level rose to 550μmol/L (Table 1).

**Figure 1:**
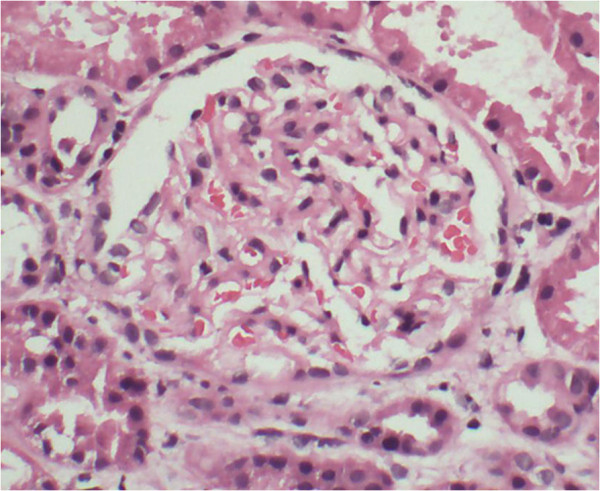
**Light microscopy of the patient’s renal biopsy tissue.** We found that 18 glomeruli were present in the sample. The glomeruli show an almost normal appearance. The capillary lumina are patent. The basement membranes are intact and not duplicated. The mesangium is not widened and not proliferated. The interstitial space shows a multifocal, mild mononuclear cell infiltration. The tubules are minimally atrophic. The vessels are unremarkable. There is no obvious chronic damage in the kidney. Hematoxylin and eosin stain; original magnification, ×200.

**Figure 2:**
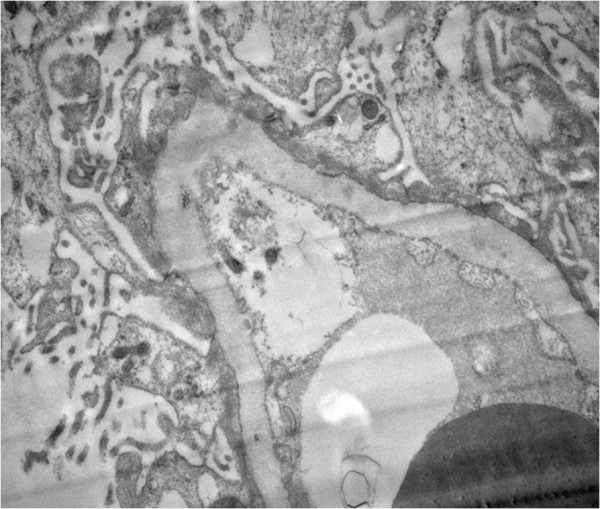
**Electron microscopy of the patient’s renal biopsy.** This section shows a marked effacement of the foot processes of podocytes as well as a microvillous transformation The mesangial area is unremarkable. The basement membrane is irregularly wrinkled. The capillary lumina are patent. No electron deposits are visible. Uranyl acetate and lead citrate double-stain; original magnification, ×15,000.

Our first diagnostic impression was severe LN. A percutaneous renal biopsy was performed on the patient’s fourth hospital day. Unexpectedly, glomeruli showed an almost normal appearance under light microscopy. The capillary lumina were patent. The basement membranes were intact and not duplicated. The mesangium was not widened and was not proliferated. The intertitium space showed a multifocal, mild mononuclear cell infiltration. The tubules were minimally atrophic. The vessels were unremarkable. There was no obvious chronic damage in the kidney (Figure 1). Immunofluorescence microscopy revealed an absence of deposits of immunoglobulin, complements and fibrinogen. By electron microscopy, we observed a marked effacement of the foot processes of podocytes as well as a microvillous transformation. The mesangial area was unremarkable. The basement membrane was irregularly wrinkled. The capillary lumina were patent. There were no electron deposits observed by electron microscopy (Figure 2). With a diagnosis of MCD, an “adequacy” dose steroid regimen (prednisolone 1mg/kg/day, 50mg/day) was administered orally. Other adjuvant therapies included hemodialysis, diuresis and supplemental plasma albumin.

**Table 1:** **Changes in multiple indicators during admission** ^**a**^

Hospital day	SCr (μmol/L)	Alb (g/L)	Urine output (ml/24 hr)	ANA	C3 (g/L)
1	455	22.8	250	1:320	0.31
4^b^	550	21.9	50	–	–
10	184	24.6	750	–	–
15	99.5	25	1500	–	–
20	58.6	26.4	2000	1:100	0.6

^a^Alb, Albumin; ANA, Antinuclear antibody; C3, Complement 3; SCr, Serum creatinine. ^b^Start of prednisolone administration.

**Figure 3:**
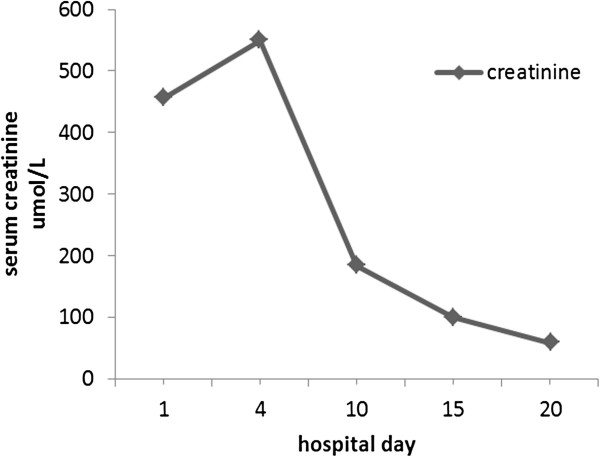
**Changes in serum creatinine levels during admission.**

A few days after the initiation of steroids, the patient’s edema and her high serum creatinine level remitted slowly (Figure 3). By the 17th day of therapy, her serum creatinine level decreased to a normal level (58.6μmol/L), and her serum albumin and urine output increased to 26.4g/L and 2000ml/24 hr, respectively (Table 1). She was discharged from the hospital with improved symptoms and signs. One month after she was discharged, laboratory investigations revealed negative urinary protein and normal serum albumin levels. The dosage of oral steroids was tapered gradually over the course of the next 10 months. After continuous treatment, her nephrotic syndrome resolved completely and no signs of relapse were noted during 2 years of follow-up.

## Discussion

SLE is a prototype autoimmune disease involving multiple organs, with the kidney being most common. This disease is known as LN. A large number of immune complex depositions in the kidney are the main reason for the onset of LN. A “full-house” phenomenon visualized under an immunofluorescence microscope and electron-dense deposits in the glomeruli are its main pathological features. According to the International Society of Nephrology and Renal Pathology Society (ISN/RPS) consensus, LN is classified into types I to VI. The nephrotic syndrome is commonly associated with diffuse, proliferative LN (ISN/RPS class IV) and membranous LN (ISN/RPS class V) [2]. Our patient had a long history of SLE; however, her renal biopsy findings and clinical course were typical of MCD.

The coexistence of AKI, MCD and SLE, as we saw in our patient, is extremely rare. Treatments for LN and MCD are different. Patients with ISN/RPS class III or IV LN should receive long-term aggressive therapy, including high doses of daily oral corticosteroids, intravenous pulses of corticosteroids, intravenous pulses of cyclophosphamide therapy, mycophenolate mofetil and cyclosporine A, among other medications [3]. However, treatments for MCD mainly involve oral corticosteroids for 6 months to 1 year, possibly in addition to other immunosuppressive therapies. The clinical diagnosis, prognosis and treatment of LN should be assessed with the support of a histological analysis, unless a renal biopsy is performed, in which case administration of a cytotoxic agent is unavoidable.

The presence of lupus and MCD in the same patient may not be just a coincidence. In the literature, 13 cases on the association between SLE and MCD have been reported (Table 2) [4–9]. Among these cases, SLE developed simultaneously with the onset of MCD in five cases and after the onset of MCD in three cases. MCD developed during the course of SLE in five patients. Among these 13 patients, four patients combined with abnormal renal function. Only one case was linked to nonsteroidal anti-inflammatory drugs (NSAIDs); the rest of the patients had no obvious secondary factors. After administration of prednisolone or NSAID discontinuation, remission was achieved in all patients. Their edema disappeared, their serum creatinine decreased to normal levels and their urinary protein turn to negative.

**Table 2:** **Literature review of patients with SLE and MCD** ^**a**^

Author	Year	Sex	Age, yr	SCr (mg/dl)	Clinical course
Matsumura *et al.* [10]	1989	F	37	n.d.	SLE developed when MCD relapsed
		F	21	n.d.	SLE developed when MCD relapsed
		F	22	n.d.	SLE developed simultaneously with onset of MCD
Okai *et al.* [5]	1992	M	22	n.d.	SLE developed simultaneously with onset of MCD
Makino *et al.* [11]	1995	F	41	1.51	MCD developed during course of SLE
Horita *et al.* [4]	1997	F	25	n.d.	SLE developed simultaneously with onset of MCD
Nishihara *et al.* [12]	1997	F	17	0.5	SLE developed when MCD was in remission
Guery *et al.* [13]	1998	F	27	3.41	SLE developed simultaneously with onset of MCD
Perakis *et al.* [14]	1998	F	45	1.1	MCD developed during course of SLE
Seo *et al.* [8]	2002	F	41	2.2	SLE developed simultaneously with onset of MCD
Wang *et al.* [7]	2006	F	19	1.9	MCD developed during course of SLE (NSAID-induced)
Hong *et al.* [6]	2011	F	24	0.6	MCD developed during course of SLE
Redondo-Pachón *et al.* [9]	2012	F	59	0.75	MCD developed during course of SLE

^a^MCD, Minimal change disease; n.d., No data SCr, serum creatinine; SLE, Systemic lupus erythematosus.

Our patient had significant proteinuria at the time of her initial diagnosis of lupus; regrettably, a renal biopsy was not performed at that time. Thus, we were unable to distinguish which lesion developed first or whether they arose simultaneously. The association between MCD and the concurrent presence of SLE is not absolutely clear. Immune abnormalities, especially T-cell dysfunction, were considered to be the common cause of SLE and MCD in our patient. Patients with SLE with a renal involvement have a low helper/suppressor T-cell ratio. In MCD, glomerular permeability factors derived from T cells, such as interleukin 13, is known to increase glomerular permeability [15]. There may be another pathogenic link, which is known to be associated with genes coded within the major histocompatibility complex between these two diseases. A question remains whether SLE simply co-occurs with MCD or is a precipitating agent of MCD.

## Conclusion

We reported a case of AKI associated with MCD in a patient with SLE. On the basis of our experience in this case, we offer two suggestions. First, a renal biopsy should be performed in patients with SLE to exclude other nephrotic diseases. Second, there may be an immunological association between SLE and MCD. Further investigation is needed to understand the relationship between SLE and MCD.

## Consent

Written informed consent was obtained from the patient for publication of this case report and any accompanying images. A copy of the written consent is available for review by the Editor-in-Chief of this journal.

## Abbreviations

AKI: Acute kidney injury
ANA: Antinuclear antibody
LN: Lupus nephritis
MCD: Minimal change disease
NSAID: Nonsteroidal anti-inflammatory drug
SLE: Systemic lupus erythematosus.
